# Editorial: Experimental approaches to the acquisition of information structure

**DOI:** 10.3389/fpsyg.2025.1607534

**Published:** 2025-06-17

**Authors:** Tania L. Leal, Bradley Hoot, Stavros Skopeteas, Joseph V. Casillas, Oksana Laleko

**Affiliations:** ^1^Department of Spanish & Portuguese, University of Arizona, Tucson, AZ, United States; ^2^Department of Modern Languages, DePaul University, Chicago, IL, United States; ^3^Institute for Linguistics, Georg-August University of Göttingen, Göttingen, Germany; ^4^Department of Spanish and Portuguese, Rutgers, The State University of New Jersey, New Brunswick, NJ, United States; ^5^Linguistics Program, State University of New York at New Paltz, New Paltz, NY, United States

**Keywords:** information structure, topic/focus preposing, experimental linguistics, language acquisition, focus

Utterances vary in terms of their (in)felicitousness depending on how constituents relate to the discourse context, speakers' communicative needs, and speakers' assessment of hearers' beliefs. The study of *information structure* explores how speakers package their utterances into blocks with varying informational values, formalizing these units with notions like “focus,” “background,” or “topic” (Krifka, 2008). These categories have received substantial interest in linguistics, generating both theoretical models and experimental studies bearing on how information structure is represented and interpreted in the minds of speakers (for overviews, see Féry and Ishihara, 2016; Krifka and Musan, 2012).

Research on information structure has boomed in recent years. Our survey of related terms on Scopus spanning 1960–2024 yielded 1,879 peer-reviewed journal articles, with the bulk of scholarship published in the past decade (Figures 1a, b)^1^.

**Figure 1:**
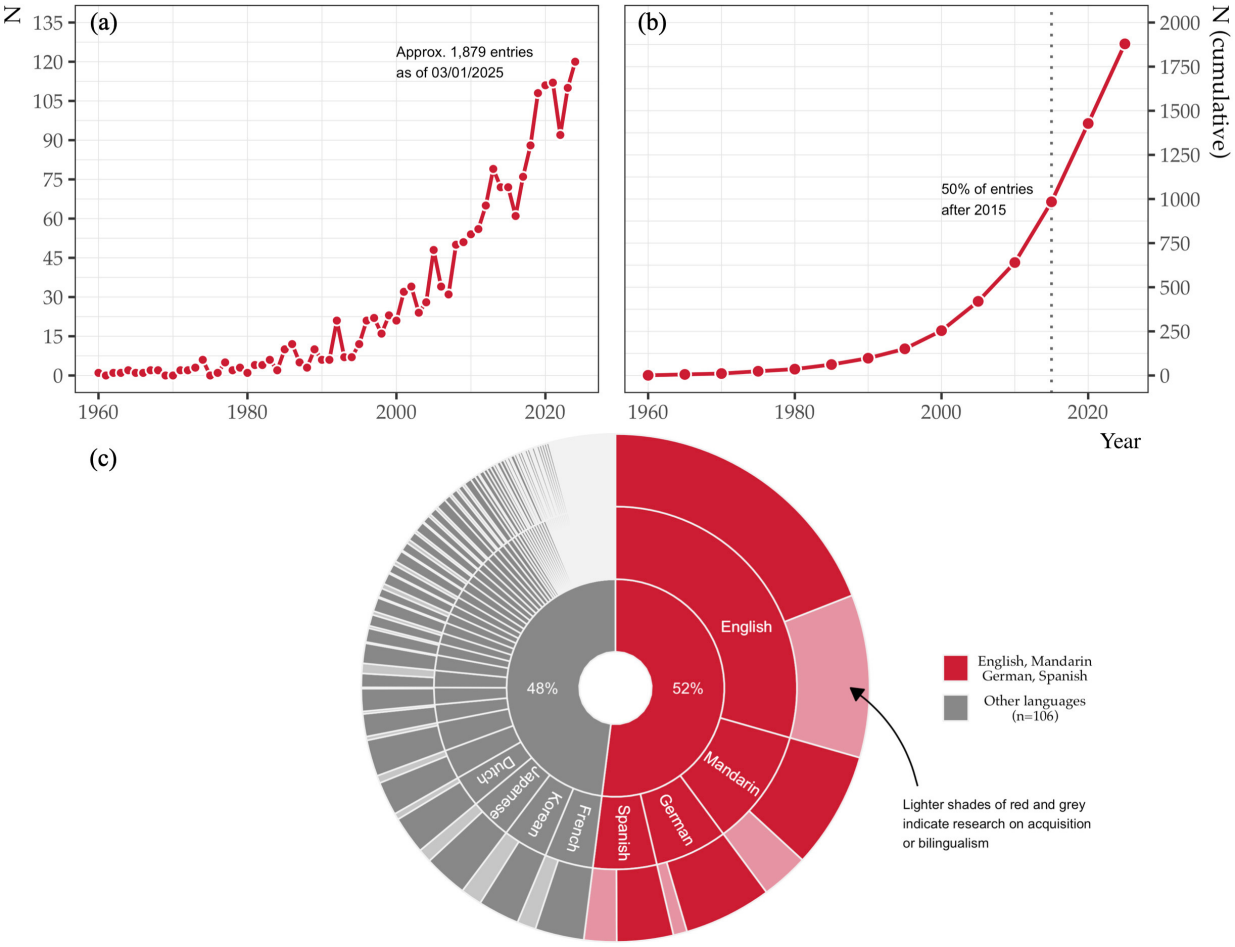
Number of articles per year **(a)** and cumulative totals in five-year intervals **(b)** featuring information structure terms in Scopus, 1960–2024. Proportion of Scopus articles on information structure by language and population, 1960–2024 **(c)**.

Despite the surge, investigating how information structure is *acquired* remains in its early stages, with uneven coverage across populations and languages. As shown in Figure 1c, the representation of individual languages in our survey follows a power-law distribution, with 52% of the articles focusing on just four languages. Only 25% of articles in our sample include at least one term related to acquisition or bilingualism (see text footnote 1).

The contributions to this Research Topic address these lacunae by expanding the cross-linguistic scope, incorporating data from child L1 acquirers, L2 and heritage bilinguals, and contexts of societal multilingualism, and utilizing both traditional and innovative methods.

Lozano and Quesada use CEDEL2 corpus texts to examine anaphora resolution in Spanish native speakers and English-speaking Spanish learners. Their findings challenge the Position of Antecedent Strategy (Carminati, 2002) as the default strategy, showing anaphora resolution is more complex than experimental data suggests, with overt pronouns rarely used and often substituted by repeated noun phrases.

Uth et al. demonstrate, using an oral production task and a corpus study, that focus in Yucatec Maya is incompatible with progressive aspect marking. Appealing to a semantic account, they argue that progressive aspect blocks focus fronting because the marker itself functions as a type of focalization.

Seraye Alseraye examines how incomplete speech representations affect processing of garden path sentences in L2 Arabic, finding faster reading times in unambiguous contexts and when disambiguating segmental information is present. Overall comprehension remained unaffected, even in the presence of incorrect disambiguating information. The study supports the “good-enough” model of language processing (Ferreira et al., 2009) among L2 learners of an understudied language.

Slioussar and Harchevnik explore how L1 Russian speakers and Mandarin Chinese L2 Russian learners process SVO and OVS word orders. Using online (reading times) and offline (sentence rating) tasks, they show that both groups benefit from given-before-new structures, although L2 learners struggle more with processing non-canonical word orders and are less sensitive to discourse constraints.

Lorenzen et al. employ a novel paradigm—an interactive reading task—to increase the ecological validity of spoken data. They examine how information status affects prosodic prominence in German, finding that paradigmatic effects appear mainly in F0, while syntagmatic effects vary across speakers and depend on the specific acoustic parameter.

Destruel et al. investigate the acquisition of French prosody using a virtual robot-mediated picture-matching task. Unlike younger children, 7- to 8-year-olds and adults use prosody to distinguish focus from non-focus. Furthermore, this study finds subject-object asymmetries, attributed to the dominant use of syntactic strategies for subject focus in French.

Yang et al. examine how young children acquire prosodic phrasing to mark focus in Korean. Using a picture-matching task, they find that children (ages 4–5) pattern like adults in distinguishing narrow from broad focus and prefocal material, but not from postfocal material or contrastive focus. By age 11, patterns are adult-like, with acquisition speed linked to form-meaning transparency.

Smeets uses two tasks to test clitic-doubled left dislocation in Romanian, which has received less attention than other Romance languages. The finding that L1 Romanian speakers who learned L2 Italian show attrition—unlike those who learned L2 English—highlights the role of L1-L2 similarity in reshaping L1 information structure via feature reassembly.

Luchkina et al. used two aural identification tasks (with and without contexts) to investigate how English-Russian heritage bilinguals process Russian non-contrastive focus, examining constituent order and prosodic cues. While higher-proficiency heritage speakers patterned with native speakers, the group overall tended to assign focus to nouns with nuclear stress in SVO orders—unlike native speakers—which highlights the challenges external interface structures pose (Sorace, 2011).

Neocleous and Sitaridou examine information-structural reflexes of contact between VO and OV languages. Romeyka, an Asia-Minor Greek variety (VO), has coexisted alongside Turkish (OV) for centuries. As a result, left peripheral configurations like focus movement occur in a wider range of contexts than in other Greek varieties.

Each article fills the literature gaps we identified, offering directions for future research to build on. At the methodological level, a key desideratum in information structure research is to improve the ecological validity of data, minimizing lab speech artifacts. Several contributions address this by proposing novel experimental designs (e.g., Lorenzen et al.) or combining experimental and observational research (e.g., Uth et al.). We envision future studies in which these avenues will be further pursued.

Another major challenge in studying information structure is disentangling the roles of different linguistic layers involved in its expression. The interplay between syntax and prosody in particular is central to several contributions. Destruel et al. examine the syntax-prosody complementarity in French focus expression, while Luchkina et al. investigate how prosodic and syntactic cues contribute to focus processing in Russian. We see a continued need for such nuanced, multi-layered approaches to the cross-linguistic inventory encoding these distinctions.

Finally, studying different populations beyond literate adult monolinguals—such as naturalistic and instructed bilinguals, L1 acquirers at different stages, and speakers of vernacular varieties—is imperative to understanding how grammars vary within and across languages. Some contributions show effects on attrition (Smeets) or adaptation under language contact (Neocleous and Sitaridou), while others reveal particular processing challenges in L2 learners and other bilinguals (Slioussar and Harchevnik). These findings enrich broader discussions on how dynamic processes like acquisition and language contact shape the representation and processing of information structure across diverse linguistic systems.

## Footnotes

^1^For the list of terms, see https://github.com/jvcasillas/acquisition_information_structure.
